# Impact of the systematic use of the volume-viscosity swallow test in patients with acute ischaemic stroke: a retrospective study

**DOI:** 10.1186/s12883-020-01733-0

**Published:** 2020-04-25

**Authors:** Zhu-Yun Liu, Xiao-Pei Zhang, Miao-Miao Mo, Ri-Chun Ye, Cai-Xia Hu, Min-Qing Jiang, Man-Qiu Lin

**Affiliations:** 1 Department of neurology, The Second Affiliated Hospital of Guangzhou University of Chinese Medicine, Guangzhou, China; 2 Third Department of neurology, The Second Affiliated Hospital of Guangzhou, University of Chinese Medicine, Guangzhou, China

**Keywords:** Dysphagia, Ischaemic stroke, Swallow screening

## Abstract

**Background:**

Dysphagia is common after stroke. Patients with dysphagia have a higher risk of stroke-associated pneumonia (SAP) and poor outcomes. Early detection of dysphagia is necessary to identify and manage patients at high risk of aspiration. The aim of the study was to assess the impact of the systematic administration of the volume-viscosity swallow test (V-VST) in patients with acute ischaemic stroke.

**Methods:**

This was a retrospective observational study that enrolled patients with acute ischaemic stroke in two consecutive time periods: pre-V-VST, when the 30-mL water-swallowing test (WST) was systematically administered, and V-VST, when all patients underwent the WST and the V-VST test was systematically administered if the patient failed the WST.

**Results:**

Two hundred and 42 patients were enrolled. The mean age of the participants was 68.8 ± 10.88 years, 61.2% were male, and the median National Institutes of Health Stroke Scale score was 3 (IQR, 1–6). A total of 147 patients were enrolled during the pre-V-VST period and 95 were enrolled during the V-VST period. There was a significant difference in the occurrence of SAP (21.8% vs. 10.5%, *p* = 0.024) and the rate of nasogastric tube feeding (25.9% vs. 14.7%, *p* = 0.040) between the two groups, and no differences were found in the length of hospital stay (*p* = 0.277) or the total cost of hospitalization (*p* = 0.846).

**Conclusions:**

The V-VST was a better clinical screening tool, and it can also provide detailed suggestions regarding dietary modifications to prevent aspiration and SAP.

## Background

Stroke is one of the leading causes of death and disability worldwide and is the first leading cause of death in China [29]. Poststroke dysphagia, or impaired swallowing, is a common complication of acute stroke and a risk factor for airway aspiration and stroke-associated pneumonia (SAP) [15]. Of patients with acute stroke, the incidence of dysphagia ranged from 37 to 78% depending on the diagnostic clinical or instrumental assessment tool used [15, 19]. SAP affects 7–33% of patients with acute stroke, especially patients with dysphagia after stroke [10, 15]. SAP is related to increased mortality and poor outcomes, placing a large financial burden on individuals and national health systems [28]. Pneumonia caused by dysphagia has estimated increased costs of $19,000 to $25,000 per occurrence and even larger costs when associated with feeding tube placement [12, 25]. Aspiration pneumonia caused by dysphagia is a potentially preventable hospital-acquired condition [1]. Early systematic dysphagia screening, detection and intervention are necessary to prevent dysphagia-related pneumonia [16].

Swallowing screening after stroke is performed to identify patients with dysphagia who are at risk of aspiration when swallowing using assessment tools with high sensitivity so that professionals can evaluate and intervene further to prevent SAP [24]. Several screening tools with different strengths and limitations have been developed for this purpose, but choosing which one to use depends on the local health resources [7].

Video-fluoroscopy (VFS) is a sensitive tool to assess dysphagia after stroke, but it is unlikely to be done in each patient after acute stroke as it is an expensive resource and not accessible in most hospitals in China [30]. Although VFS is the gold standard for dysphagia screening [6], a simple and sensitive bedside screening tool is needed for the acute phase in patients with stroke. The water-swallowing test (WST) is a cost-effective bedside screening tool to detect dysphagia in clinical practice [2]. The sensitivity of WST for aspiration ranged from 34.8 to 55.7%, and the specificity ranged from 78.9 to 93.2% [18]. However, WST is limited in its accuracy, as it assesses patients only based on 30 mL of water swallowing. Patients should adapt the volumes and viscosity of food boluses according to their dysphagia levels, which cannot be determined by WST [17].

The volume–viscosity swallow test (V-VST) is a bedside screening method that uses food boluses with different volumes and viscosities. The V-VST can assess patients’ swallowing functions with food boluses of different volumes and viscosities, which may be more ideal than the WST [4, 21, 27]. The V-VST is based on reducing the volume and increasing the viscosity of the bolus. According to the outcomes, the appropriate volume and viscosity of food boluses for stroke patients to minimize the risk of aspiration and SAP can be recommended [21, 23]. Previous studies have shown that the V-VST had ideal sensitivity and specificity for detecting dysphagia [4, 22]. The sensitivity of V-VST was 69% for residue, 88% for piecemeal deglutition, and 85% for identifying patients whose deglutition improved by enhancing bolus viscosity, and the specificity for these parameters was 81, 88, and 74%, respectively [4]. Rofes [22] also demonstrated that the V-VST showed 94% sensitivity and 88% specificity for dysphagia, 79% sensitivity and 75% specificity for impaired efficacy, and 87% sensitivity and 81% specificity for impaired safety.

Limited evidence has shown that V-VST is reliable for the detection and diagnosis of dysphagia in patients with acute/chronic stroke. Our study aimed to compare the clinical impact of detecting dysphagia using two bedside dysphagia screening tests in acute ischaemic stroke patients.

## Methods

### Study design

This is a retrospective observational study. All participants were evaluated for swallowing function within 6 h after their admission. Patients were screened by the WST or the WST first followed by the V-VST. All other data, including demographics and clinical features, were recorded and analysed.

### Participants

Participants were eligible if they received a diagnosis of acute ischaemic stroke that was confirmed by Computerized Tomography (CT) or Magnetic Resonance Imaging (MRI), were aged > 18 years, did not have a history of disease that can cause dysphagia, and were assessed within 14 days after stroke onset. We excluded patients who had tracheal intubation and a Glasgow Coma Scale score of < 10 (excluded if motor function was < 6 or response was < 3).

### Procedure

The participants were allocated to the pre-V-VST group or the V-VST group according to the different screening methods used during the two time periods. During the pre-V-VST period (February 2017–June 2017), all patients were systematically screened for dysphagia by a 30-mL WST to determine the existence of dysphagia. The 30-mL WST was routinely administered in the department. In the V-VST period (July 2017–October 2017), all patients were routinely screened by the 30-mL WST within 6 h after admission before they consumed food.

Two trained nurses administered the two swallowing tests to all patients who had a stroke. The two nurses were clinical nurse specialists in the stroke unit, and they received professional training regarding the basic theory and common methods of screening for dysphagia. To use the V-VST method systematically and scientifically, they went to the speech treatment centre to receive training from speech therapists for a week. The content of the training included the knowledge and practice of the V-VST. At the end of the study, the nurses received a comprehensive real-situation examination by two senior speech therapists that included the screening steps for the V-VST, explanation to patients, interpretation of screening results, and the formulation of dietary recommendations. Those who passed the examination could screen patients by the V-VST in the stroke unit. The vital signs were monitored during the test.

The WST procedure was as follows: Each patient was asked to drink 30 mL of water without interruption. Nurses observe the time of swallow and any signs of choking or coughing. Level I indicated that the patient could swallow the water in one gulp and without choking or coughing in 5 s. Patients experienced choking or coughing or failed to drink the water in one gulp were considered to have failed the test [18]. Level II indicated that patients could swallow the water in two or more gulps without choking or coughing in 5 s and needed dietary guidance [3]. The adapted dietary plan was decided by a multidiscipline team of physicians, nurses and therapists, and the plan included improving food viscosity, regulating eating position, and controlling eating speed and eating methods. Level III indicated that patients could swallow 30 mL of water in one gulp but with coughing or choking, and level IV indicated that patients could swallow the water in two or more gulps with coughing or choking. Level V indicated that patients could not swallow 30 mL of water with frequent coughing or choking (Table 1). Levels III to V were indicators of tube feeding [3]. Level I was defined as negative for dysphagia, and levels II to V were defined as positive for dysphagia.

**Table 1:** Methods, criteria for interpretation, and intervention guidance of WST

Levels	Criteria	Outcome and guidance
Level I	Patients can swallow the water in one gulp and without choking or coughing in 5 s	Negative Can eat through mouth safely and effectively
Level II	Patients can swallow the water in two or more gulps without choking or coughing in 5 s	Positive Can eat through mouth with dietary guidance
Level III	Patients can swallow the water in one gulp but with coughing or choking	Positive Indicators of tube feeding
Level IV	Patients can swallow the water in two or more gulps with coughing or choking	
Level V	Patients cannot swallow 30 mL of water with frequent coughing or choking	

Methods: Each patient was asked to drink 30 mL of water in sitting or fowler position without interruption. Nurses observe the time of swallow and any signs of choking or coughing

*WST* water swallowing test

**Fig. 1:**
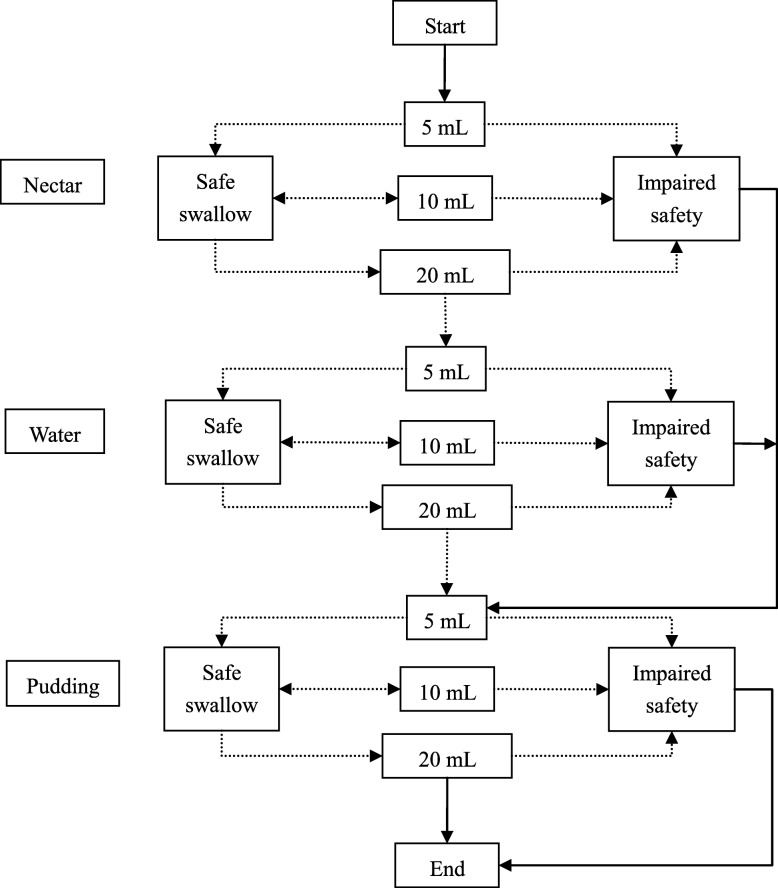
V-VST algorithm. Patients with safe swallow started the exploration with a 5 mL nectar bolus, followed by 10 and 20 mL nectar boluses, then performed the water with increasing volume and finally completed the pathway with the three water boluses to explore efficacy of swallow. If the patient presented signs of impaired safety at nectar or water viscosities, the series was interrupted and the pudding series was assessed. V-VST, volume-viscosity swallowing test

The V-VST procedure was as follows: The patient’s swallowing function was assessed at the patient’s bedside by using increasing volumes of 5, 10 and 15 ml food boluses with different viscosities of water, nectar, and pudding [4, 21]. The nectar viscosity was achieved by adding 6.4 g ThickenUp (Nestlé Health Science) to 140 mL water. The pudding viscosity was achieved by adding 12.8 g ThickenUp to 140 mL water. All boluses of each volume and viscosity were offered to the patients with a syringe by a proficient nurse. The V-VST measured the maximum volume of one gulp that a patient could achieve safely and effectively. The test started at nectar viscosity in increasing volume, and if any signs of reduced safety were presented, the test continued with the pudding viscosity and omitted the liquid viscosity tests. However, if no concerns for safety were observed, the test was continued with liquid viscosity in increasing volumes. Finally, the pudding viscosity with increasing volume was tested (Fig. 1). Any signs of impaired safety or effectiveness were recorded during screening.

Signs of impaired safety included alterations in voice tone, coughing during or after eating, or a decrease in oxygen saturation of more than 3% compared to baseline. Signs of impaired efficacy included impaired labial seal (inability to maintain the whole bolus in the oral cavity after swallowing), oral residue (presence of part of the bolus in the cavity after swallowing), pharyngeal residue (auto-reported by the patient as the feeling of having the bolus stuck in the throat after swallowing), and repeated swallowing (multiple swallows per bolus). One or more signs of impaired safety or efficacy indicated that the patient was positive for dysphagia. The screening outcomes indicated the safe and effective volume and viscosity of food boluses, helping professionals develop individualized diet plans for patients. For the patients who were assessed as having impaired safety or effectiveness at volumes less than 10 mL or whose nutrient intake was less than 60% of the intake target, tube feeding was recommended [3].

During the V-VST period, patients who achieved level I in the WST were defined as safe and effective swallowers and did not perform the V-VST, and patients who achieved level II-V in the WST continued with the V-VST. The patients who achieved safe and effective results on the V-VST were defined as negative for dysphagia, and those who showed any signs of impaired safety, impaired effectiveness, or both were defined as positive for dysphagia.

### Measures

A questionnaire on demographics and clinical stroke status was used to collect information regarding sex, age, stroke aetiology, stroke severity, presence of dysphagia, presence of hypertension, diabetes mellitus, dyslipidaemia, and coronary heart disease or previous myocardial infarction. Data on nasogastric tube feeding, occurrence of SAP, and total cost of hospitalization were also collected. Patient diagnoses were retrieved from medical records.

The severity of stroke was assessed by the NIHSS, with higher scores indicating a high level of severity [8]. SAP was defined as the presence of three or more of the following manifestations: fever ≥38 °C, productive cough, abnormal respiratory examination (tachypnoea [> 22/min], tachycardia, inspiratory crackles, bronchial breathing), arterial hypoxemia (PO2 < 70 mmHg), elevation of blood inflammatory markers and microbiological identification of a relevant pathogen, and the presence of pulmonary consolidation or infiltration in chest imaging [14].

#### Statistical analysis

Continuous variables are presented as the mean and SD, and categorical variables are expressed as frequencies (%). Independent sample t-tests and Mann-Whitney *U* tests were used for the analysis of continuous variables, and chi-square tests or Fisher’s exact tests were used for qualitative variables. Multivariate logistic regression analyses were performed to explore the predictors of SAP. All *p* values were estimated from two-tailed tests. Differences were considered statistically significant at *p* < 0.05. All data were analysed using SPSS 22.0 for Windows (SPSS Inc., Chicago, IL, USA).

## Results

**Fig. 2:**
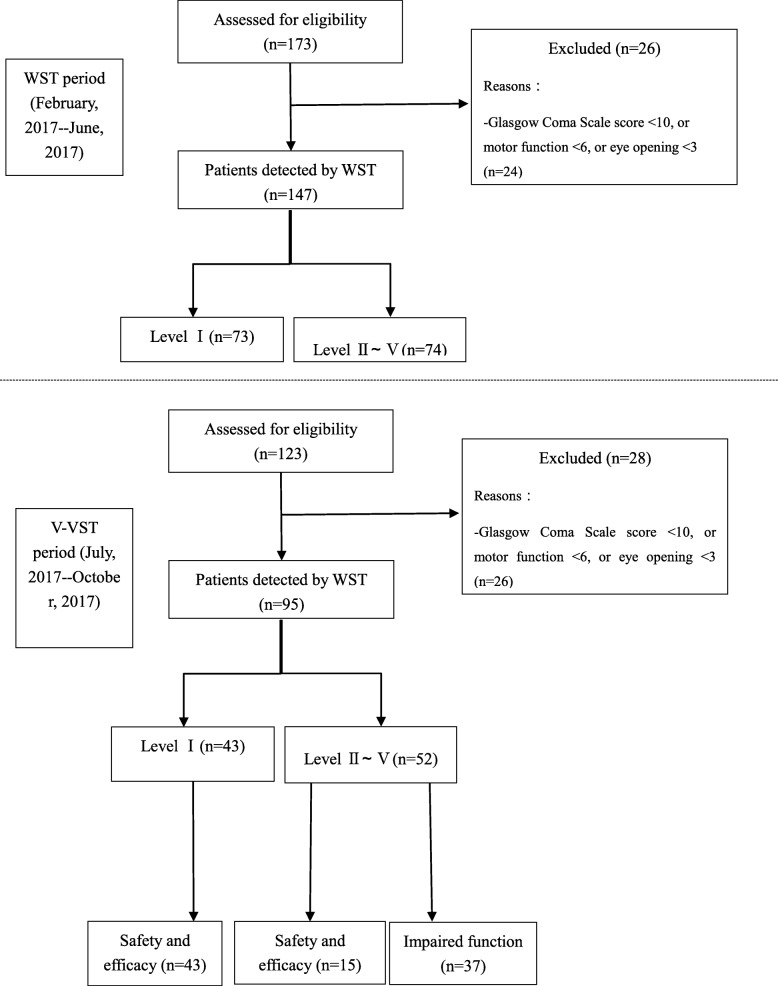
The work flow of dysphagia screening in participants

During the recruitment period, 296 patients were enrolled. Of these, 242 met the inclusion criteria, and 54 were excluded (Fig. 2). Table 2 presents the baseline characteristics of the study population according to the inclusion period. Comparison of baseline data showed no significant differences between the two groups (*p* > 0.05). During the study period, a total of 242 patients with acute ischaemic stroke were enrolled, 147 of whom were enrolled during the pre-V-VST period and 95 of whom were enrolled during the V-VST period. The mean age was 68.8 ± 10.88 (range 47–95) years, and 148 of 242 patients were male (61.2%). The median NIHSS score was 3 (IQR, 1–6). Most strokes (56.2%) were located in the partial anterior circulation, as defined by the Oxfordshire Community Stroke Project (OCSP) classification. In addition, 219 (90.5%) patients only received drug treatment.

**Table 2:** Characteristics of the study population according to the inclusion period (*n* = 242)

Variables	Pre-V-VST period (***n*** = 147)	V-VST period (***n*** = 95)	***t/Z /χ***^***2***^	***P***
**Age, years old**	69.73 ± 10.81	67.36 ± 10.88	1.67	0.097
**Gender, males**	93(63.3)	55(57.9)	0.701	0.307
**Risk factors**				
Hypertension	106(72.1)	73(76.8)	0.671	0.413
Diabetes	52(35.4)	39(41.1)	0.793	0.373
Dyslipidemia	46(31.3)	29(30.5)	0.016	0.900
Coronary heart disease/previous myocardial infraction	47(32.0)	26(27.4)	0.581	0.446
Smoking	43(29.3)	31(32.6)	0.311	0.577
Drinking	22(15.0)	15(15.8)	0.030	0.862
**Admission NIHSS score**	3(2–7)	3(1–5)	−1.643	0.100
**Admission BP, mmHg**				
Systolic BP	158.82 ± 22.80	157.83 ± 24.84	0.319	0.750
Diastolic BP	85.95 ± 13.07	88.84 ± 14.73	−1.601	0.111
**Stroke etiology**			7.328	0.086^a^
Cardioembolism	1(0.7)	5(5.3)		
Large-vessel disease	86(58.5)	57(60.0)		
Small-vessel disease	53(36.1)	30(31.6)		
Other causes	7(4.8)	2(2.1)		
Cryptogenic	0(0)	1(1.1)		
**OCSP classification**			3.610	0.307
Total anterior circulation infract	28(19.0)	11(11.6)		
Partial anterior circulation infract	80(54.4)	56(58.9)		
Lacunar infarct	22(15.0)	12(12.6)		
Posterior circulation	17(11.6)	16(16.8)		
**Treatment protocols**			3.270	0.071
Drugs	129(87.8)	90(94.7)		
Non-drugs	18(12.2)	5(5.3)		

^*^The difference was statistically significant

^a^Fisher’s exact test

Results are presented as n (%) or median (interquartile range). *NIHSS* National Institutes of Health Stroke Scale; *OCSP* Oxfordshire Community Stroke Project; *SAP* stroke-associated pneumonia; *BI* Barthel index; *BP* blood pressure

Drugs in treatment protocol means oral drugs or intravenous drugs therapy. Oral drugs therapy includes anticoagulants (Aspirin, Clopidogrel) and antithrombotic agents (Statins). Intravenous drugs therapy means administrating thrombolytic (rt—PA) within four and a half hours after stroke onset. Non-drugs in treatment protocol means endovascular interventional treatment, including thrombectomy and endovascular stent implantation

**Fig. 3:**
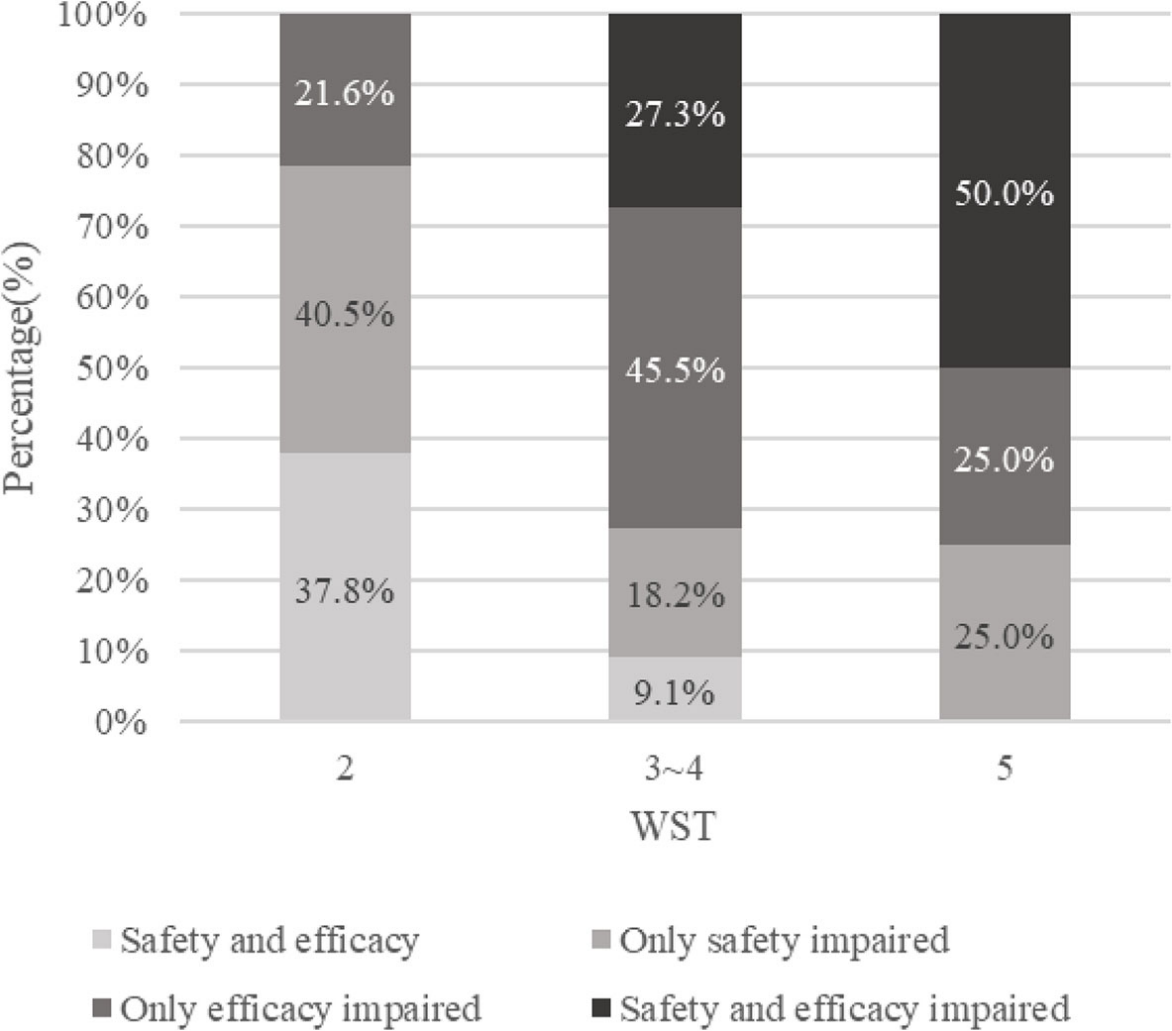
The comparison of levels of dysphagia recognized by V-VST and WST. All patients failed water test had a V-VST. Among them, 15 out of 52 were identified to have safe and effective swallowing capability. Eighteen had only impaired swallowing safety and 14 had only impaired swallowing effectiveness. Five had impaired swallowing safety and effectiveness. Patients with impaired safety or effectiveness received adjustment their food viscosity. V-VST, volume–viscosity swallow test; WST, water swallow test

Among the 95 patients in the V-VST period, 43 patients achieved level I in WST, which was considered to indicate safe and effective swallowing. All patients who failed the WST (level II-V) were then assessed by the V-VST. Dysphagia defined by the 30-mL WST was found in 74 of 147 patients (50.3%) during the pre-V-VST period. During the V-VST period, 52 of 95 (54.7%) were defined as having dysphagia by the WST, and 37 of them were defined as having dysphagia by the V-VST further (37 of 95, 38.9%). Eighteen of the 52 patients who failed during the WST were identified as only safety-impaired, 14 of 52 were identified as only efficacy-impaired, and 5 of 52 were identified as having neither impaired safety nor efficacy by the V-VST. The distribution of the V-VST results among patients who failed the WST is shown in Fig. 3. Patients with impaired swallowing safety or effectiveness screened by the V-VST received a diet with adjustment in food viscosity or volume of one bolus and avoided tube feeding.

Table 3 shows the comparison of outcomes between the WST and V-VST in patients with dysphagia. In the two inclusion periods, there were no differences in the length of hospital stay (*p* = 0.277) or total cost of hospital stay (*p* = 0.846). The SAP occurrence between the pre-V-VST and V-VST periods was significantly different (21.8% vs. 10.5%, *p* = 0.024), and the rate of nasogastric tube feeding of the V-VST group was significantly lower than that of the pre-V-VST group (14.7% vs. 25.9%, *p* = 0.040).

**Table 3:** The outcomes related with dysphagia screening according to the inclusion period (n = 242)

Variables	Pre-V-VST period (***n*** = 147)	V-VST period (***n*** = 95)	***Z /χ***^***2***^	***P***
**Nasogastric tube feeding**	**38(25.9)**	**14(14.7)**	**4.225**	0.040^*^
Length of tube feeding of patients with tube, days	11(5–20)	12(6–15.5)	−1.322	0.186
**SAP**	**32(21.8)**	**10(10.5)**	**5.085**	0.024^*^
**Nutrition index**				
Hemoglobin(g/L)	121(93–140)	121(89–139)	−0.614	0.539
Albumin(g/L)	36.6(28.1–39.5)	38.4(28.3–41.9)	1.872	0.061
Length of stay, days	11(8–15)	11(8–14)	−1.086	0.277
**Total cost, USD**	2807.8(1951.4–4461.5)	2899.4(2012.9–5074.7)	0.195	0.846
**Outcome of WST**			0.447	0.504
Negative	73(49.7)	43(45.3)		
Positive	74(50.3)	52(54.7)		

^*^The difference was statistically significant

Results are presented as n (%) or median (interquartile range)

*SAP* stroke-associated pneumonia; *WST* water swallow test; *V-VST* volume–viscosity swallow test

Table 4 shows that among patients who were defined as dysphagia positive (II-V level) in the WST during both periods, the patients who underwent the V-VST were less likely to be fed with tubes (25.0% vs. 45.9%, *p* = 0.017) and had a lower occurrence of SAP (17.3% vs. 35.1%, *p* = 0.028).

**Table 4:** The comparison of different outcomes of dysphagia by WST and V-VST in participant with II-V level by WST (*n* = 126)

Variables	Pre-V-VST period	V-VST period	***χ***^***2***^	***P***
	(***n*** = 74)	(***n*** = 52)		
**Nasogastric tube feeding**	**34(45.9)**	**13(25.0)**	**5.729**	0.017^*^
**SAP**	**26(35.1)**	**9(17.3)**	**4.838**	0.028^*^

^*^The difference was statistically significant

*SAP* stroke-associated pneumonia; *WST* water swallow test; *V-VST* volume–viscosity swallow test. In the pre-V-VST period, patients only received 30-ml WST. In the V-VST period, patients received 30-ml WST first, those achieved level II-V in WST continued with V-VST

Table 5 shows that both tests had good predictive ability for patients with pneumonia (*p* < 0.001 in the WST and *p* < 0.001 in the V-VST). During the pre-V-VST period, 26 of 74 (35.1%) patients with dysphagia developed SAP, whereas only 6 of 73 (8.2%) patients without dysphagia developed SAP (*p* < 0.001). During the V-VST period, 9 of 37 (24.3%) patients with dysphagia developed SAP, and only 1 of 58 (1.7%) patients without dysphagia developed SAP (*p* = 0.001). Both tests showed that patients who were defined as having dysphagia (positive in two tests) by WST or V-VST were more likely to be fed with a nasogastric tube, and the total cost of hospitalization and occurrence of SAP were higher.

**Table 5:** The comparison of different outcomes of dysphagia by WST and V-VST in ischemic stroke patients (*n* = 242)

Variables Gruops		Nasogastric tube	Length of stay, days	Total cost, USD	SAP
	**Positive** **(*n*** **= 74)**	34(45.9)	11.5(8–16.25)	3338.1(2171.2–7982.3)	26(35.1)
**WST(n = 147)**	**Negative** **(*n*** **= 73)**	4(5.5)	11(8.5–14)	2579.7(1859.9–3651.2)	6(8.2)
	***t/Z /χ***^***2***^	31.394	0.727	2.627	15.633
	***P***	< 0.001^*^	0.467	0.009^*^	< 0.001^*^
	**Positive** **(*n*** **= 37)**	13(35.1)	12(8–16)	3586.1(2250.9–7517.9)	9(24.3)
**V-VST(n = 95)**	**Negative** **(*n*** **= 58)**	1(1.7)	10.5(7.75–14.0)	2665.9(1858.9–4360.9)	1(1.7)
	***t/Z /χ***^***2***^	20.069	0.984	2.076	
	***P***	< 0.001^*^	0.325	0.038^*^	0.001^*a^

^*^The difference was statistically significant

^a^Fisher’s exact test

Table 6 shows that the patients of V-VST group were less likely to develop SAP compared with patients of pre-V-VST group [OR = 0.423, 95% CI (0.197, 0.907)], and the patients with tube feeding were more likely to develop SAP compared with patients without tube feeding [OR = 16.236, 95% CI (7.365, 35.792)].

**Table 6:** Multivariable binomial logistic regression with SAP as the dependent variable among ischemic stroke patients (*n* = 242)

Variables	***B***	***Wald***	***P***	***Adjusted OR***	***95% C.I.***	
					***Lower***	***Upper***
**Nasogastric tube feeding**						
Yes	2.787	47.760	< 0.001^*^	16.236	7.365	35.792
No	Reference					
**Exposure periods**						
V-VST period	−0.861	4.885	0.027^*^	0.423	0.197	0.907
Pre-V-VST period	Reference					

Adjusted for outcome of WST, outcome of V-VST

*SAP* stroke-associated pneumonia; *WST* water swallow test; *V-VST* volume–viscosity swallow test

*The difference was statistically significant

## Discussion

Dysphagia is common after stroke, and swallowing screening using an effective and accessible tool before oral intake is important for post-stroke care [20]. The prevalence of dysphagia assessed by WST was 52.1% (126 of 242), which was in accordance with other studies in which a similar screening test was used [13]. The prevalence of dysphagia assessed by the V-VST in our study was 38.9% (37 of 95), which was similar to the result of the original study [11, 13]. Likewise, the prevalence of SAP was similar to that reported in the literature [30] and was significantly higher in patients with dysphagia or at risk of aspiration, which has also been previously demonstrated [9, 13].

However, there has been no unanimous recommendation regarding the screening tool, and there are few studies comparing the benefits of different screening tools or whether the usage of a specific screening tool has a clear benefit on outcomes such as SAP [26]. Our study implemented a systematic structured bedside swallowing screening test (V-VST). There was a significant difference in the occurrence of SAP and nasogastric tube feeding in patients with acute ischaemic stroke when compared with that when 30 ml WST systematically administered. Moreover, among the patients who were defined as positive (II-V level) by the 30-ml WST, the occurrence of SAP and nasogastric tube feeding in the V-VST group was significantly lower than that in the pre-V-VST group. The rates of tube feeding and SAP of the V-VST group were significantly lower than those of the pre-V-VST group, but the length of hospitalization and overall healthcare expenditures between the two groups were not significantly different.

The WST is the most widely recognized bedside screening tool for patients with acute stroke, as it is a convenient and easier procedure. However, consuming food is different from swallowing water. Stroke patients can usually consume food with a certain consistency safely but cannot swallow water or liquid food safely [18]. The WST is limited because it could only be used to confirm whether the patient can safely swallow water [2]. Some of the patients assessed as level of III or IV by WST may swallow food boluses with nectar or pudding viscosity safely, which means that those patients can avoid tube feeding and the associated discomfort. During the acute phase of a stroke, it is important for early recovery that the volume and viscosity of boluses be adjusted based on the patient’s safety and effectiveness when swallowing [17].

In our study, the V-VST provided more information on volume and viscosity, which indicated that patients could intake foods or pills through the mouth in a safer way. The V-VST could also help professionals select the appropriate diet for patients and minimize the risk of complications [21, 23]. As the V-VST provides detailed information, professionals could address swallowing problems more precisely in patients with acute ischaemic stroke based on a bedside test. Compared with the WST, the V-VST can also be administered by nurses and reduce the rate of tube feeding in patients who can tolerate any diet [30]. In the future, the V-VST can be widely used in clinical settings to screen populations at high risk of dysphagia and prevent the occurrence of SAP by modifying the volume or viscosity of boluses.

Dysphagia screening is the first step in preventing SAP, and it can help nurses identify high-risk populations and provide chances for professionals to systematically manage them to prevent the occurrence of SAP. The prevention of SAP is comprehensive, not only relying on the identification of dysphagia by nurses but also requiring careful evaluation by speech specialists and scientific care plan by nurses. Because pneumonia could still occur even with modification of the volume and viscosity of boluses, transient nasogastric tube feeding may be implemented. This study found that nasogastric tube feeding was a predictor of development of SAP. However, many other important factors, including head position, getting up from bed, continuous tube feeding, caregivers’ experience in feeding, aspiration caused by vomiting or gastroesophageal reflux, oral hygiene, immunosuppression after stroke, and the lesion location of dysphagia, are also associated with the occurrence of SAP and must be taken into account in care plans to prevent respiratory infections in patients with acute ischaemic stroke [5]. In addition, stroke is progressive, and some patients who do not have swallowing problems at the early stage may develop dysphagia later. Dynamic swallowing screening is necessary.

Our study has several limitations. We only compared the results assessed by the WST and V-VST and did not systematically evaluate patients by VFS. VFS is not available and relatively expensive for most Chinese patients. Second, only 52 patients underwent both the WST and V-VST during the V-VST period. This relatively small number of subjects can introduce bias in the comparative assessment. The data were retrospectively collected, which may have led to some bias. However, we demonstrated the benefits of different convenient and cheaper tools for dysphagia bedside screening, and the isolated implementation of a specific dysphagia screening tool (V-VST) has a clear benefit in regard to SAP. Further well-designed research into the validation of this convenient clinical tool would add value to the clinical field. More importantly, dynamic swallow assessment and integration of screening results of V-VST into clinical decision-making and clinical care and rehabilitation plan is warranted.

## Conclusions

The assessment of dysphagia is necessary for pneumonia prevention after ischaemic stroke. The V-VST provided more detailed information on the severity of dysphagia than WST. The occurrence of SAP in patients with acute ischaemic stroke in the V-VST group was significantly lower than that in the pre-V-VST group. The V-VST may be more useful as a bedside screening tool since it helps guide dietary management and adaptations for dysphagia.

## Abbreviations

CT: Computerized Tomography
MRI: Magnetic Resonance Imaging
SAP: Stroke-associated pneumonia
VFS: Video-fluoroscopy
V-VST: Volume viscosity swallow test
WST: Water swallowing test
